# SIGMAR1 variants in ALS–PD complex cases: a case report of a novel mutation and literature review

**DOI:** 10.3389/fneur.2023.1242472

**Published:** 2023-09-13

**Authors:** Haining Li, Tingting Xuan, Ting Xu, Juan Yang, Jiang Cheng, Zhenhai Wang

**Affiliations:** ^1^Department of Neurology, General Hospital of Ningxia Medical University, Yinchuan, China; ^2^Diagnosis and Treatment Engineering Technology Research Center of Nervous System Disease of Ningxia Hui Autonomous Region, Yinchuan, China; ^3^School of Clinical Medicine, Ningxia Medical University, Yinchuan, China; ^4^Department of Neural Electrophysiology, Cardiovascular and Cerebrovascular Disease Hospital, General Hospital of Ningxia Medical University, Yinchuan, China; ^5^Institute of Medical Sciences, General Hospital of Ningxia Medical University, Yinchuan, Ningxia, China; ^6^Ningxia Engineering Technology Research Center for Diagnosis and Treatment of Nervous System Diseases, Neurology Center, General Hospital of Ningxia Medical University, Yinchuan, Ningxia, China

**Keywords:** amyotrophic lateral sclerosis, Parkinson’s disease, *SIGMAR1*, genotype, phenotype

## Abstract

Amyotrophic lateral sclerosis (ALS) is a devastating neurodegenerative disease characterized by progressive degeneration of upper and lower motor neurons, with occasional involvement of the extrapyramidal system. Mutations in the sigma non-opioid intracellular receptor 1 (*SIGMAR1*) gene have been identified as one of the causes of ALS. Here, we present a case of a 49-year-old man diagnosed with ALS–Parkinson’s disease (PD) complex. The patient exhibited bradykinesia and tremor, and whole-exome sequencing revealed homozygous mutations in the *SIGMAR1* gene (c.446-2A > T). In addition, we conducted an investigation into the clinical and molecular phenotype of previously reported variants of *SIGMAR1* associated with ALS. This case report aims to raise awareness among physicians regarding atypical phenotypes of amyotrophic lateral sclerosis and to encourage further research on the factors leading to *SIGMAR1* mutations in patients.

## Introduction

Amyotrophic lateral sclerosis (ALS) represents the most common form of motor neuron disease, characterized by the degeneration of upper and lower motor neurons. ALS is classified into two types: familial (fALS) and sporadic (sALS), with the latter accounting for 90–95% of cases, while fALS comprises only 5–10%. The etiology of sALS remains largely unknown although it is believed to involve both genetic and environmental factors. Genetic factors, in particular, play a significant role in the occurrence of sALS. To date, more than a hundred ALS-related genes have been identified, with approximately 30 genes primarily associated with ALS (1). Among Asians, Cu/Zn superoxide dismutase (SOD1) gene mutations are the most prevalent, whereas pathogenic mutations in the sigma non-opioid intracellular receptor 1 (*SIGMAR1*) gene are rare in Asian patients with familial or sporadic ALS. While mutations in the *SIGMAR1* gene have been reported in association with ALS, with or without frontotemporal dementia or juvenile ALS, no instances of this mutation in the ALS–Parkinson’s disease (PD) complex have been described until now. Here, we present the case of a 49-year-old patient with ALS–PD complex, exhibiting slowly progressing motoneuron disease that may be linked to a homozygous *SIGMAR1* mutation. Additionally, we conduct a comprehensive review of cases of ALS patients with mutations in this gene, as reported in the relevant scientific literature.

## Case report

**Figure 1:**
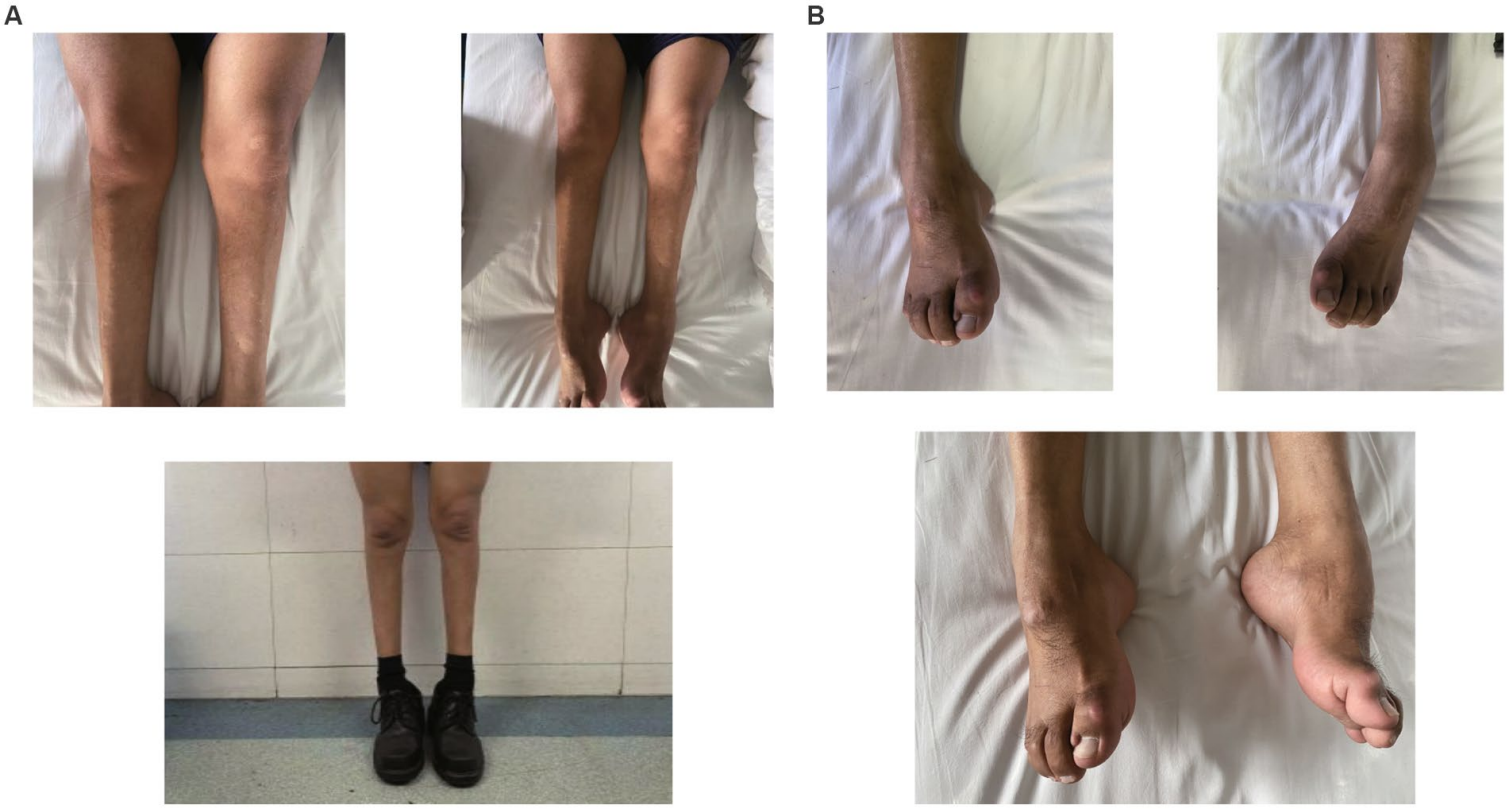
**(A)** Atrophy of bilateral calves more pronounced distally giving a “stork leg” appearance akin to Charcot-Marie-Tooth disease. **(B)** Planovalgus deformities of both feet.

**Figure 2:**
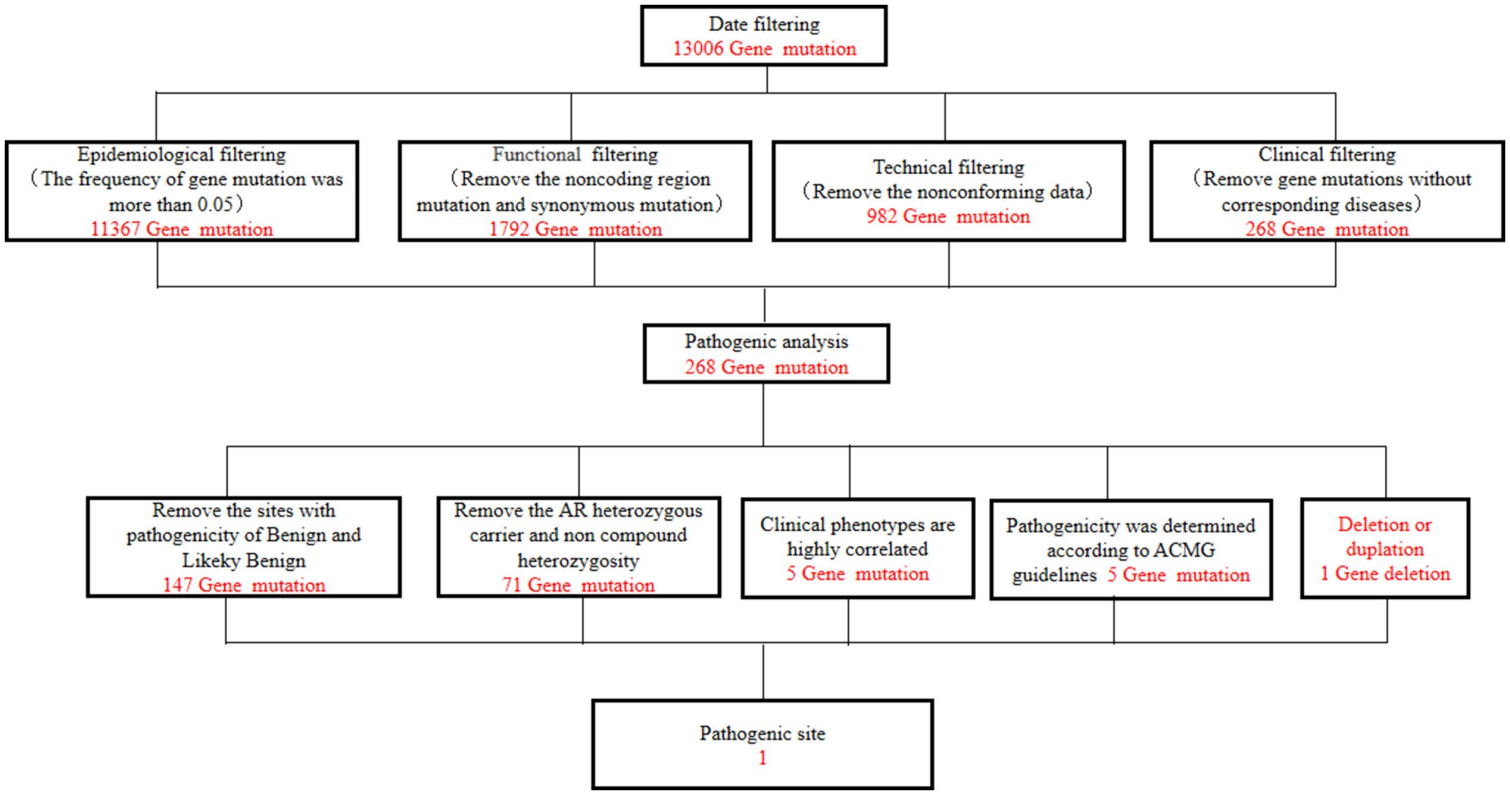
Filtering steps for the variant calls in whole-exome sequencing.

**Figure 3:**
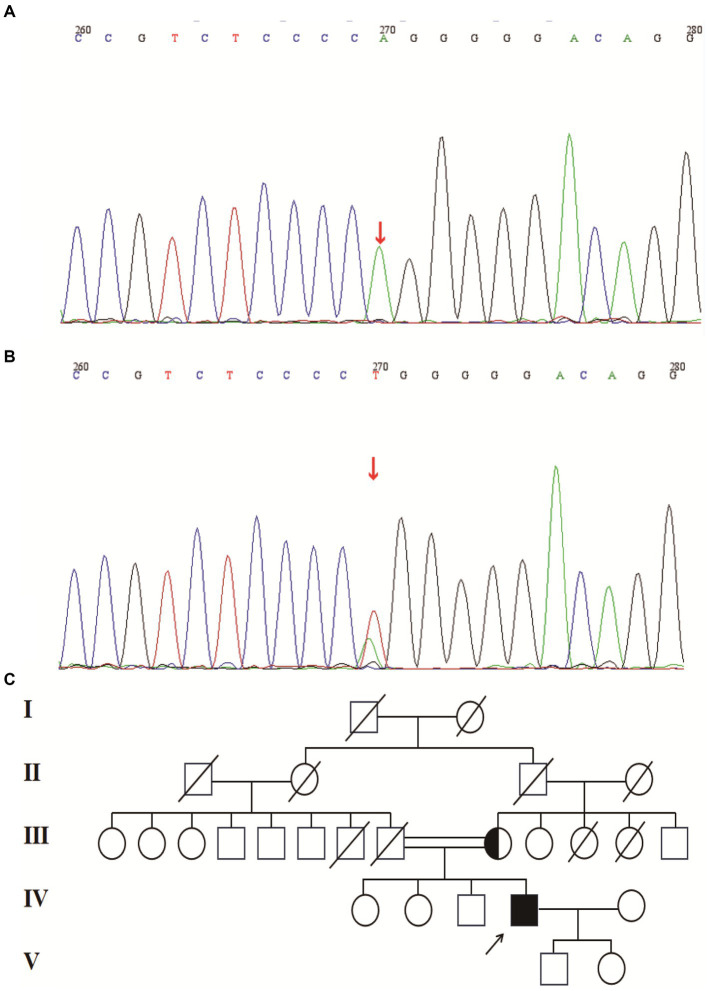
**(A)** Splice site mutation in the SIGMAR1 identified in our patient. Sanger sequencing was performed using cDNA generated from the patient. **(B)** Mutation in the SIGMAR1 identified in our patient’s mother. **(C)** Mutant pedigree map of familial mutations. Circles = females; squares = males; and slashes = deceased.

A 49-year-old man presented to our neurology department with complaints of involuntary shaking in both upper limbs for the past 3 years, along with slowness of movement for the past 2 years. He exhibited rest and action tremors in both upper limbs, along with simultaneous occurrence of bradykinesia and rigidity. Subsequently, he experienced unresponsiveness, memory decline, and choking while drinking, and his speaking rate began to slow down. Additionally, his facial expressions started to diminish, as noticed by his wife. In April 2021, the patient received a diagnosis of Parkinsonian syndrome at a local hospital. Initial treatment with levodopa at a daily dosage of 100 mg, gradually increased to 200 mg, resulted in partial improvement in involuntary shaking, but showed no significant overall improvement. Over the next year, his symptoms rapidly worsened, with progressive aggravation of stiffness and the appearance of mental irritability. Neurological examinations revealed decreased spontaneous facial expressions, poor eye movement in all directions, horizontal nystagmus, mildly increased muscle tone in the neck and limbs, and deep tendon reflexes in the biceps and triceps (1+). A positive Babinski sign was observed bilaterally. Symmetric muscle atrophy of the calves was also noted, which he reported experiencing for as long as he could remember (Figure 1A). Additionally, it was noted that he had planovalgus deformities of both feet since the age of 5 years (Figure 1B), a condition similar to that of his uncle’s feet. The strength of his upper and lower extremities, as well as proximal and distal muscles, was assessed as 5 on the Medical Research Council Muscle Scale. Brain magnetic resonance imaging revealed only mild atrophy, and his cognitive functions were deemed normal, scoring 28 on the standardized Mini-Mental State Examination and 23 points on the Montreal Cognitive Assessment. Further cervical and thoracic spine MRI showed degenerative changes, and electromyography revealed chronic denervation in both upper and lower extremities. Motor nerve conduction studies demonstrated reduced conduction velocity, amplitude, and distal latency in the left median nerve, as well as in the bilateral tibial and peroneal nerves. Sensory nerve conduction testing revealed normal sensory nerve action potential but showed delayed F-wave latencies in the left median and tibial nerves. However, anal sphincter electromyography was normal. The somatosensory-evoked potential showed abnormalities in the bilateral lower limbs, indicating a conduction block in the somatosensory pathway from the spinal cord to the cortex. Moreover, the bilateral visually evoked potential and bilateral auditory brainstem response were also found to be abnormal. The visually evoked potential showed prolonged P100 latency in both eyes. The auditory brainstem response suggested that bilateral ears were stimulated, but the waveform on both sides was relatively poor. The ambulatory electroencephalogram monitoring was normal. The routine cerebrospinal fluid (CSF) analysis showed normal pressure, cell counts, and levels of protein and glucose. Finally, whole-exome sequencing was performed using MyGenostics. In this study, four steps were employed to select potential pathogenic mutations for downstream analysis: (i) mutations with read counts less than 5 and mutation ratios below 30% were excluded; (ii) mutations with a frequency greater than 5% in 1,000 g, ESP6500, and Inhouse databases were removed; (iii) mutations present in the InNormal database (MyGenostics) were also discarded; (iv) synonymous mutations not listed in the HGMD database were excluded. The remaining mutations were considered potential pathogenic mutations for further analysis (Figure 2). Genomic DNA was extracted from the patient’s whole blood, and subsequent sequencing analysis identified a novel splice site mutation in intron 3 of SIGMAR1 gene (c.446-2A>T), which was further confirmed by Sanger sequencing (Figures 2, 3A). A review of the patient’s medical history revealed a longstanding presence of planovalgus deformities in both feet for over 40 years. Physical examination revealed muscle atrophy of both lower limbs at 10 years old, and he complained of mild discomfort while walking. However, his general condition was normal. The patient did not pursue further examination or treatment at that time. During the current clinical examination, upper and lower motor neuron damage was observed, and all the above findings were consistent with the diagnosis of ALS. At that time, the Unified Parkinson’s Disease Rating Scale-Part III motor score (in the morning without antiparkinsonian therapy) was 40. Next, we conducted a levodopa load test, and he showed a good response to levodopa. Based on the findings, the patient was eventually diagnosed with ALS–PD complex. Further exploration of the patient’s family history revealed that his parents were close relatives as they were second cousins. Unfortunately, his father was dead. Genetic testing was conducted on the mother, and it revealed that she has the same SIGMAR1 variant as detected in the proband (Figure 3B). Subsequently, a pedigree analysis was performed (Figure 3C). At the 3-month follow-up examination, the symptoms were observed to have remained relatively stable.

**Table 1:** Clinical phenotypes of patients with SIGMAR1 mutations.

References	Source	Clinical diagnosis	Nucleotide change	Phenotype	Initial symptoms	Mode of inheritance	Age of onset (years or median years)
Al-Saif et al. (2)	Saudi Arabia	ALS	c.304G>C	fALS	Lower limb spasticity and weakness	AR	(1.5)
Luty et al. (3)	Australia	ALS-FTLD/FTD	c.672*51G>T	fALS	NA	AD	NA
Ullah et al. (4)	Pakistani	ALS	c.672*31A>G	fALS	Progressive weakness of the upper limbs	AR	30
Izumi et al. (5)	Japan	ALS	c.505T>A c.622C>T	sALS	Slowly progressive gait disturbance	NA	80
Karasozen et al. (6).	USA	ALS	c.283dupC	sALS	An orthopedist for toe walking	Uniparental disomy	4
Kim et al. (7)	Korea	ALS	c.58T>C	sALS	Slowly progressive limb weakness	NA	47
Tripolszki et al. (1)	Hungary	ALS	c.125T>G	sALS	Tetraparesis	NA	(67.5)

ALS, amyotrophic lateral sclerosis; fALS, familial ALS; sALS, sporadic ALS; AR, autosomal recessive; AD, autosomal dominant; NA, no information available.

**Figure 4:**
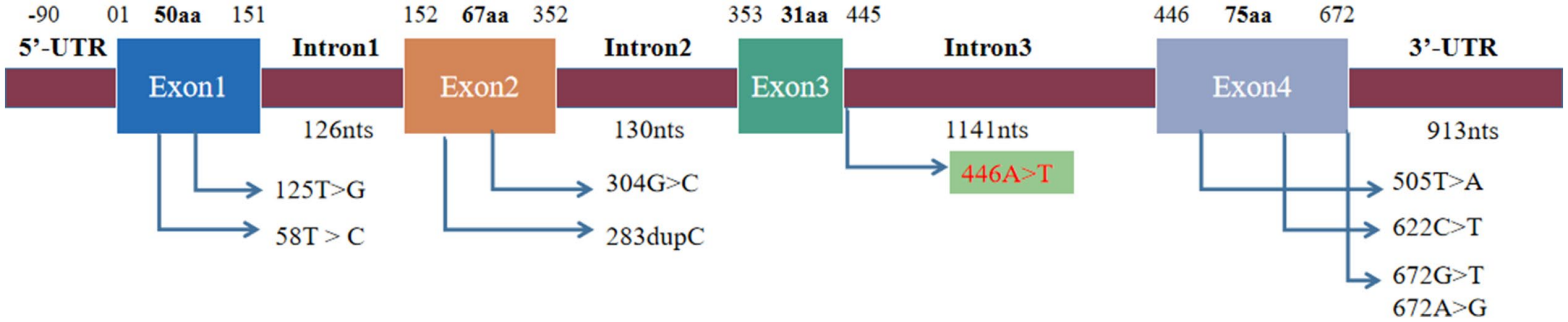
Mutation site of SIGMR1. The previously reported mutation site was located in exons and 3′-UTR, and the mutation site was located in intron.

## Review of previously reported cases of ALS patients with SIGMAR1 mutations

A literature review was conducted by searching PubMed and China National Knowledge Infrastructure (CNKI) databases from their inception until May 2023 using the keywords “*SIGMAR1*,” “ALS,” and “amyotrophic lateral sclerosis.” Relevant articles describing cases of ALS with *SIGMAR1* mutations were selected. Among the articles, eight described studies of interest, three reported cases of familial ALS (fALS), and five reported cases of sporadic ALS (sALS). Various mutations in the *SIGMAR1* gene were identified in affected individuals, including a mutation (c.67251G > T) in the 3′-untranslated region in the FTLD-MND pedigree and mutations (c.304G > C, c.67231A > G, c.505 T > A, c.622C > T, c.283dupC, c.58 T > C, c.125 > G) in the *SIGMAR1* gene in affected ALS patients. The phenotypes of each of these cases are presented in Table 1 and Figure 4.

## Discussion and conclusion

According to the revised El Escorial criteria, patients with ALS may exhibit extrapyramidal involvement in addition to upper and lower motor neuron symptoms and signs (8). When ALS is associated with PD, it is known as Brait–Fahn–Schwartz syndrome or ALS–PD complex. Parkinsonism in these patients typically resembles PD and shows a response to levodopa. ALS–parkinsonism is more common than ALS–PD complex and refers to the presence of extrapyramidal findings that do not respond to levodopa treatment in ALS patients (8). Mutations in the *SIGMAR1* gene have previously been linked to different forms of ALS, including juvenile (onset age < 20 years old) and adult-onset (early onset within 20–60 years and late onset >60 years) cases (9). Most SIGMAR1-related ALS cases present with a typical ALS phenotype, but the SIGMAR1 c.672*51G > T variant was identified in an Australian familial FTLD cohort with ALS, presenting as an ALS-frontotemporal dementia (FTD) phenotype (3). In this report, we present a case of ALS–PD complex in a patient with juvenile onset. We identified a potentially new pathogenic variant (c.446-2A > T) located in intron 3 of the *SIGMAR1* gene. The sequencing data was saved in FASTQ format, and MGI sequencing adapters and low-quality reads (<80 bp) were filtered using the cutadapt software.^1^ The clean reads were then mapped to the UCSC hg19 human reference genome using the BWA parameter of the Sentieon software.^2^ Next, we removed duplicated reads using the driver parameter of Sentieon software, which also corrected the base to improve the quality of the output BAM file reads, making them closer to the real probability of mismatch with the reference genome. The mapped reads were used for variation detection. Variants of SNP and InDel were identified using the driver parameter of the Sentieon software. The data were then transformed into VCF format. To further analyze the variants, we used ANNOVAR software^3^ to annotate and cross-reference multiple databases, including 1,000 genome, ESP6500, dbSNP, EXAC, Inhouse (MyGenostics), HGMD, SIFT, PolyPhen-2, MutationTaster, and GERP++. Based on the ACMG guidelines, this variant was predicted to be likely pathogenic (PVS1+PP3+PM2). To the best of our knowledge, this is the first reported case of *SIGMAR1*-related ALS presenting as ALS–PD complex.

Compared to previously reported cases, the patient in this report exhibited distinct clinical phenotypes. While muscle weakness is the most commonly observed clinical feature in patients with *SIGMAR1* mutations (10), the patient in our case presented with no symptoms of muscle weakness but instead displayed extrapyramidal symptoms, including bradykinesia, rigidity, and tremor. It is noteworthy that in the majority of ALS patients, motor nerve conduction velocities and terminal latencies are normal. However, this particular patient showed decreased conduction velocity, amplitude, and distal latency in the left median nerve, bilateral tibial, and peroneal nerves, which may be particularly relevant to the long-term atrophy of the lower limbs. Through our literature review, we found cases of ALS patients with *SIGMAR1* mutations presenting with ALS either with or without frontotemporal dementia as well as cases of juvenile ALS. Nevertheless, this is the first reported case, where an association between ALS–PD phenotype and *SIGMAR1* mutations has been observed.

*SIGMAR1* is comprised of four exons and three introns, located on chromosome 9p13.3. This gene exhibits ubiquitous expression in various human tissues, including the brain (cerebellum), retinal ganglion cells, astrocytes, liver, and placenta. Particularly, it is prominently expressed in motor neurons found in the brainstem and spinal cord. The *SIGMAR1* gene plays diverse roles in different cells and organs, encompassing ion channel regulation, chaperone function, regulation of mitochondrial morphology, dynamics, and function, as well as involvement in autophagy and endoplasmic reticulum (ER) stress response (10, 11). A common pathological feature in ALS is the disruption of the ER-associated membrane, where the ER-resident chaperone protein is predominantly localized (12, 13). Consequently, changes in *SIGMAR1* function may alter ER morphology and impact ER stress responses, resulting in abnormal mitochondrial damage and the initiation of cellular autophagy, thereby contributing to the pathogenesis of ALS. PD, another prevalent neurodegenerative disease, has also been associated with alterations in *SIGMAR1* functions. Studies by Finsterer et al. (9) demonstrated that *SIGMAR1* agonist treatment in mice with 6-hydroxydopamine lesions reduced neuroinflammation, increased the density of dopaminergic fibers in denervated striatal regions, and elevated the levels of neurotrophic factors. Furthermore, Hong et al. (14) revealed that *SIGMAR1* deficiency reduced 1-methyl-4-phenyl-1,2,3,6-tetrahydropyridine-induced death of dopaminergic neurons and parkinsonism. Thus, pharmacological activation/inhibition of *SIGMAR1* may potentially slow down the progression of PD. Overall, *SIGMAR1* activation has demonstrated protective effects in neurodegenerative diseases by modulating various cellular pathways, including the regulation of mitochondrial function, autophagy, calcium homeostasis, and chaperone function.

*SIGMAR1* activation has been found to induce potent neuroprotective effects, promote neuronal survival, and restore neuronal plasticity, leading to a deceleration of disease progression in neurodegenerative conditions. Moreover, it has shown promise in ameliorating the clinical symptoms of these diseases. On the contrary, dysfunction of *SIGMAR1* may exacerbate the advancement of neurodegenerative disorders. Positioned at the interface of two crucial organelles commonly implicated in the majority of neurodegenerative disorders—the mitochondria and the ER—*SIGMAR1* emerges as a robust therapeutic target with significant potential for intervention.

## Data availability statement

The datasets presented in this article are not readily available because of ethical and privacy restrictions. Requests to access the datasets should be directed to the corresponding authors.

## Ethics statement

The studies involving human participants were reviewed and approved by the General Hospital of Ningxia Medical University. The patients/participants provided their written informed consent to participate in this study. Written informed consent was obtained from the individual(s) for the publication of any potentially identifiable images or data included in this article.

## Author contributions

TXua examined the patient clinically. JY performed and analyzed neuroradiologic imaging studies. TXu analyzed electromyography results. HL analyzed performed genetic analyses. HL and TXua wrote the manuscript. JC and ZW analyzed and interpreted the data and revised the manuscript. All authors contributed to the article and approved the submitted version.

## Funding

This research study was supported by the Key Research and Development Program of Ningxia Hui Autonomous Region (grant number 2022BEG03130), the Natural Science Fund project in Ningxia (grant number 2022AAC03561), and the National Nature Science Foundation of China (grant number 81960245).

## Conflict of interest

The authors declare that the research was conducted in the absence of any commercial or financial relationships that could be construed as a potential conflict of interest.

## Publisher’s note

All claims expressed in this article are solely those of the authors and do not necessarily represent those of their affiliated organizations, or those of the publisher, the editors and the reviewers. Any product that may be evaluated in this article, or claim that may be made by its manufacturer, is not guaranteed or endorsed by the publisher.

## Footnotes

^1^ http://code.google.com/p/cutadapt/

^2^ https://www.sentieon.com/

^3^ http://annovar.openbioinformatics.org/en/latest/
